# Polycyclic Aromatic Hydrocarbons Content in Contaminated Forest Soils with Different Humus Types

**DOI:** 10.1007/s11270-018-3857-3

**Published:** 2018-06-09

**Authors:** Jarosław Lasota, Ewa Błońska

**Affiliations:** Department of Forest Soil Science, Faculty of Forestry, University of Agriculture, Al. 29 Listopada 46, 31-425 Krakow, Poland

**Keywords:** Anthropogenic factors, Mor and mull humus types, PAHs, Soil organic matter

## Abstract

The aim of the study was to determine polycyclic aromatic hydrocarbon (PAH) content in different forest humus types. The investigation was carried out in Chrzanów Forest District in southern Poland. Twenty research plots with different humus types (mor and mull) were selected. The samples for analysis were taken after litter horizons removing from a depth of 0–10 cm (from the Of- and Oh-horizon total or A-horizon). pH, organic carbon and total nitrogen content, base cations, acidity, and heavy metal content were determined. In the natural moisture state, the activity of dehydrogenase was determined. The study included the determination of PAH content. The conducted research confirms strong contamination of study soil by PAHs and heavy metals. Our experiment provided evidence that different forest humus types accumulate different PAH amounts. The highest content of PAHs and heavy metals was recorded in mor humus type. The content of PAHs in forest humus horizon depends on the content and quality of soil organic matter. Weaker degradation of hydrocarbons is associated with lower biological activity of soils. The mull humus type showed lower content of PAHs and at the same time the highest biological activity confirmed by high dehydrogenase activity.

## Introduction

Polycyclic aromatic hydrocarbons (PAHs) are chemical substances with carcinogenic and mutagenic potential (Srogi 2007). PAHs originate mainly from combustion processes (industry, heating) as well as from mobile sources (vehicular emissions) (Krauss et al. 2005, Shen et al., 2013). PAHs in the soil are characterized by low mobility and high durability (Maliszewska-Kordybach et al. 2008). The content and composition of polycyclic aromatic hydrocarbons in the soils are the result of processes occurring in them. The course of these processes depends on the properties of soils (temperature, pH, soil organic matter content) (Aichner et al. 2015). Soils appear to act as a sink for PAHs, due to the high affinity of those compounds to soil organic matter (SOM) (Sweetman et al. 2005).

The type of stand in the forest area considerably affects the content of PAHs. Błońska et al. (2016) recorded a higher content of aromatic hydrocarbons in the mineral horizons of soils of deciduous stands. According to Horstmann and McLachlan (1998), the canopy of trees increases pollutant fluxes from air to soil, by litter fall. Additionally, species composition of trees affects soil organic matter (Augusto et al. 2002; Hobbie et al. 2007). Soil organic matter affects the physical and chemical properties of the soil and its overall health. Fekete et al. (2012) showed that increasing detritus input enhances the stimulating effect of soil moisture content on microbial activity. The enzyme activity was significantly related to the light fraction carbon suggesting that enzymes respond to pools that are more immediately degradable and unprocessed (Veres et al. 2015). According to Yang et al. (2010a), sequestration in SOM was critical for PAH distribution in soils. The lower degradation rate in the soil with higher SOC is the major reason for the association between SOC and PAH concentrations (Yang et al. 2010b). The strong adsorption of hydrophobic organic chemicals to SOC can delay the global redistribution of organic pollutants (Ockenden et al. 2003). According to Syed et al. (2017), PAH content in forest soils is mainly associated with local biomass burning and/or coal combustion. Forests have been suggested to play an important role in global distribution and fate of organic pollutants.

Previous studies evaluating the amount of PAHs in soils concern urban environments, grasslands, and agriculture areas (Aichner et al., 2015; Zhang et al. 2012; Jiao et al. 2017). There is little data on polycyclic aromatic hydrocarbons content in forest soils. The area covered by the study is located near a cluster of onerous industrial plants, including mines, a power plant, metallurgical plants and a refinery included in the Upper Silesian Industrial Region. The aim of the study was to demonstrate the amount of PAHs in forest soils with various types of humus and to complete the knowledge of soil organic matter effect on PAH distribution in forest soils. Mor and mull humus types were included in the study. Additionally, the activity of dehydrogenases was applied in order to reflect the microbial activity of forest humus contaminated with polycyclic aromatic hydrocarbons.

## Materials and Methods

### Study Area

The study was carried out in the south of Poland in the Chrzanów Forest District (50° 7′ 18 N; 19° 31′ 29 E). The study plots were located in one compact forest complex. The study area is located in the industrial emission zone, having its source in the Upper Silesian Industrial Region. The study area covered 100 ha. The study area is dominated by stagnosols (WRB (World Reference Base For Soil Resource), 2014), created on water and glacial formations. The average annual temperature for this area is 7.8 °C, and the average annual rainfall is 658 mm. The pedunculate oak (*Quercus robur*), Scots pine (*Pinus sylvestris*), and silver birch (*Betula pendula*) play the dominant role in stands. The selection of research plots was made on the basis of soil and stands maps. The final selection of the study plots was made during field observation. On each study plots, the small pit soil was dug and the horizons sequence was verified. Soil samples for laboratory analysis were collected in August 2016. Twenty study plots, ten plots with mull-type humus, and ten plots with mor-type humus were selected for the study. The first group of plots (mull-type humus) was dominated by oak-pine-birch mixed stands. Mor-type humus plots were associated with pine stands. The samples for analysis were collected after removing the litter horizon from a depth of 0–10 cm (from horizons Of and Oh in total, or horizon A). In all the cases, the samples for the study were collected from four sub-stands of soil. For determination of dehydrogenase activity and PAH content, fresh samples of natural moisture were sieved through a sieve (ø 2 mm) and stored at 4 °C in the dark before analysis.

### Laboratory Analysis of Soil

Soil samples obtained in the field were dried and sieved through 2.0-mm mesh. The pH of the samples was analyzed in H_2_O and KCl using the potentiometric method. The content of carbon (C) and nitrogen (N) was measured with an elemental analyzer (LECO CNS TrueMac Analyzer, Leco, St. Joseph, MI, USA). The concentration of cations and content of Cd, Cr, Cu, Ni, Pb, and Zn was determined by an ICP (ICP-OES Thermo iCAP 6500 DUO, Thermo Fisher Scientific, Cambridge, UK). The sum of base cations (BC) was calculated. The content of Cd, Cr, Cu, Ni, Pb, and Zn was determined after prior mineralization in the mixture of concentrated nitric acid and perchloric at the ratio 2:1.

Dehydrogenase activity (DHA) was determined by the reduction of 2,3,5-triphenyltetrazolium chloride (TTC) to triphenyl formazan (TPF) using Lenhard’s method according to the Casida procedure (Alef, 1995).

Ten grams of soil was weighted from each sample, and then polycyclic aromatic hydrocarbons were extracted from these amounts with 70 ml of 2-propanol. The samples were centrifuged (4500, 5 min); the supernatant was collected. The supernatants were extracted to solid phase (5 ml/min)—solid-phase extraction (Chromabond Cn/SiOH). The residue was dissolved in acetonitrile and analyzed with the HPLC method. The Dionex UltiMate 3000 HPLC system was equipped with a fluorescence detector (FLD) and Dionex UltiMate 3000 Column Compartment—C18 5 μm, 4.6 × 100-mm HPLC column. The mobile phase was (a) water and (b) acetonitrile at a flow rate of 1 ml/min. From the standard PAH Calibration Mix (CRM 47940) at a concentration of 10 μg/ml, the calibration solutions at different concentrations (i.e., 0.1, 0.2, 0.5, 1, 2 μg/ml) were prepared. Each of the prepared solutions was dispensed to a chromatography column—obtained chromatograms designated the calibration curve. Then, the soil samples in triplicate were dispensed. After each ninth analysis, the “unknown” sample (calibration solution with a concentration 0.1 μg/ml) which was control material was injected. Fourteen priority PAHs were determined: naphthalene (Nft), fluorene (Flu), acenaphthylene (Acy), phenanthrene (Phe), anthracene (Ant), fluoranthene (Flt), pyrene (Pyr), benzo(a)anthracene (BaA), chrysene (Chr), benzo(k)fluoranthene (BkF), benzo(b)fluoranthene (BbF), benzo(a)pyrene (BaP), indeno(1,2,3-c,d)pyren (IcdP), and bezo(ghi)perylene (BghiP).

### Statistical Analysis

In order to reduce the number of variables in the statistical data set and to visualize the multivariate data set as a system of coordinates in a high-dimensional data space, the principal component analysis (PCA) method was applied. The PCA method was also used in order to interpret other factors, depending on the type of data set. In PCA analysis, the chemical properties, enzymes activity of soil, the sum of PAHs, and type of humus were used. Properties of different humus type were compared using a parametric honestly significant difference (HSD) test. The Pearson correlation coefficients between PAH content and chemical characteristics of humus were also calculated. The statistical significance of the results was verified at the significance level of alpha = 0.05. All the statistical analyses were performed with Statistica 12 software (2012).

## Results

**Fig. 1:**
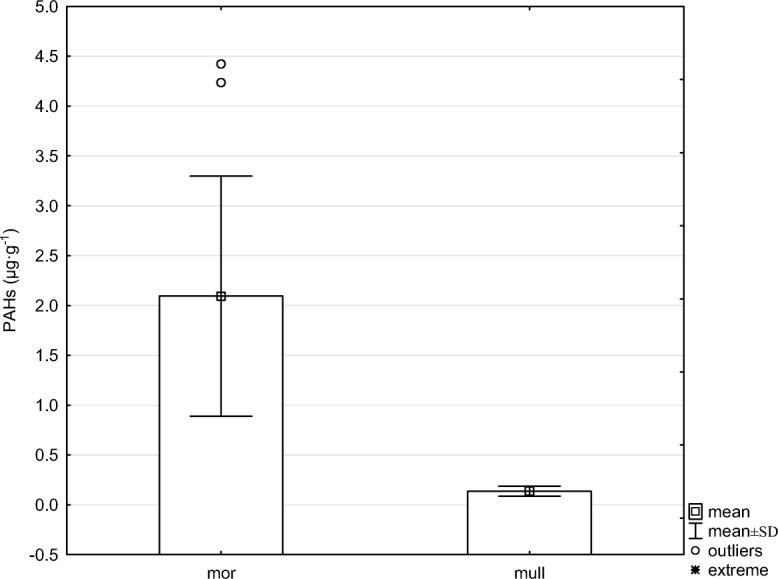
Mean sum of PAHs content (μg g^−1^) in different humus types

**Fig. 2:**
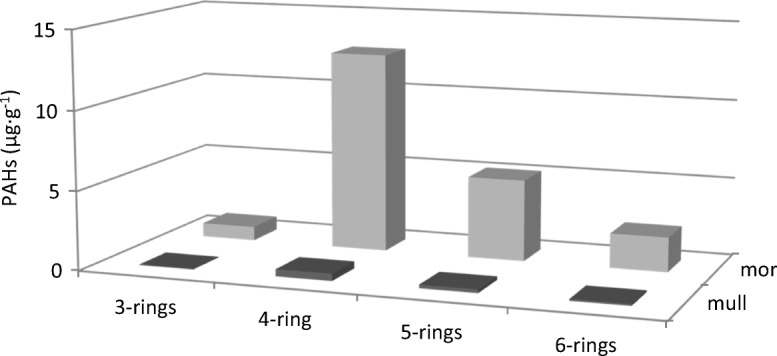
PAHs content in different humus types depending on the benzene rings

**Fig. 3:**
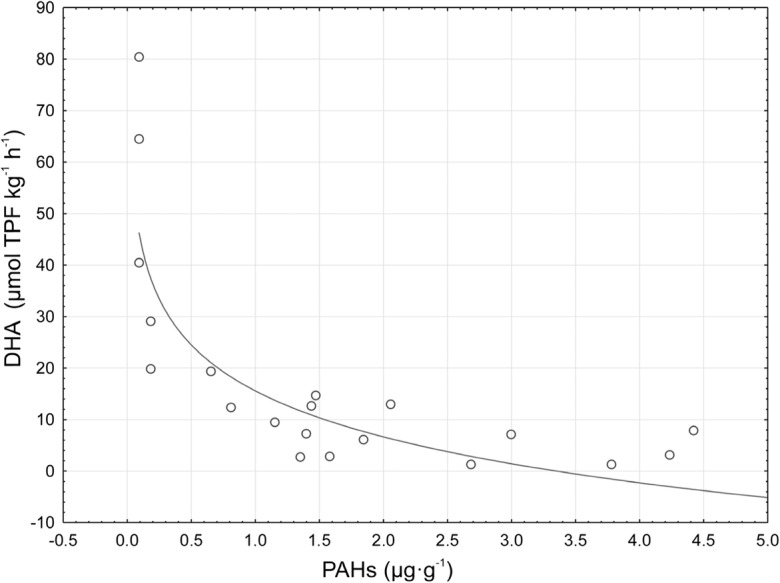
Relationships between PAHs content and dehydrogenase activity (DHA)

**Fig. 4:**
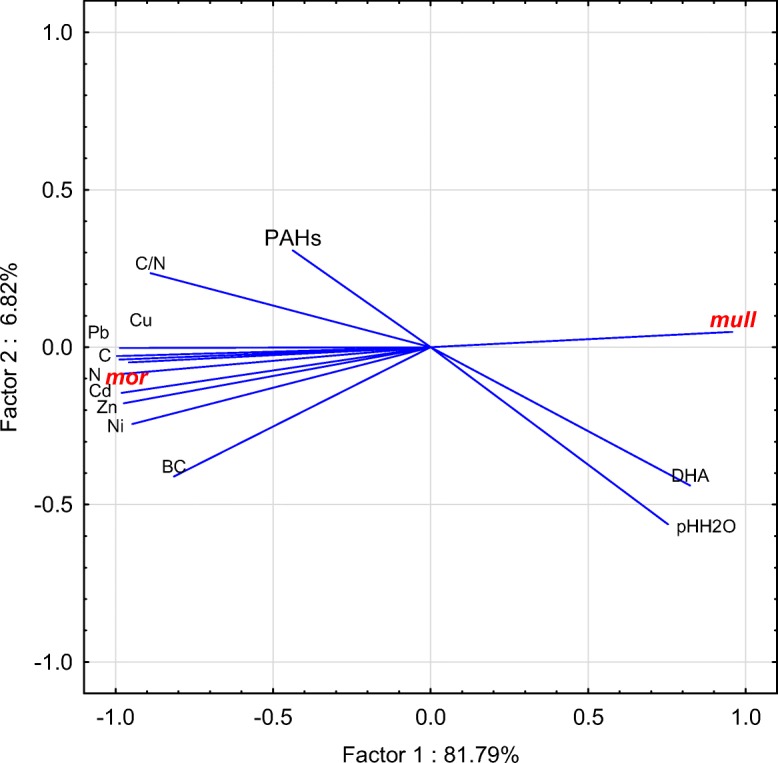
Projection of the variables on the factor plane in different humus types (mor and mull); PAHs sum of polycyclic aromatic hydrocarbons, BC base cation content, DHA dehydrogenase activity

The examined forest humus samples differed in chemical properties (Table 1). The average pH in H_2_O value of mull-type humus was 4.14 and it was 3.81 in case of mor-type humus. Statistically significant differences in all examined properties, except pH in H_2_O, were noted between the mull- and mor-type humus (Table 1). Statistically significantly higher content of C and N was noted in mor-type humus; the average content of C and N in mor-type humus was 35.98 and 1.47%, respectively. Statistically significantly higher content of cations was noted in mor-type humus. BC of mor humus was 6.65, and for mull humus, it was 2.87. Two- to threefold statistically significantly higher content of particular heavy metals was noted in mor-type humus (Table 1). Very high levels of Pb and Zn were found in the examined samples. The content of lead in mor-type humus was 389.83 mg kg^−1^, and in mull-type humus, it was 3.5-fold lower and amounted to 111.21 mg kg^−1^. In the case of zinc, its content in mor-type humus was 174.41 mg kg^−1^, and in mull-type humus, it was 2.5-fold lower and amounted to 68.43 mg kg^−1^. Depending on the type of humus, the average dehydrogenase activity ranged from 7.99 to 41.12 μmol TPF kg^−1^ h^−1^. Statistically significantly higher (fivefold) dehydrogenase activity was noted in mull-type humus (Table 1). Figure 1 shows the average sum of polycyclic aromatic hydrocarbons content in particular types of humus. A clearly higher content of PAHs was noted in mor-type humus. Regardless of the number of rings, the higher content of particular hydrocarbons was noted in mor-type humus (Fig. 2). Four and 5 ring hydrocarbons predominated in the examined humus. The lowest content was recorded for PAHs with three rings. Significant correlations between PAH content and chemical properties were noted (Table 2). PAH content significantly negatively correlated with pH and dehydrogenase activity (Fig. 3). The content of PAHs was positively correlated with the level of C and N and heavy metal content. A projection of the variables on the factor plane clearly demonstrated correlations between the properties and type of humus. Two main factors had a significant total impact (88.6%) on the variance of the variables. Factor 1 explained 81.79% of the variance of the examined properties, whereas factor 2 accounted for 6.82% of the variance (Fig. 4). Figure 4 shows a high PAHs and heavy metal content in mor-type humus. Mull-type humus is associated with an increased dehydrogenase activity and pH. PCA confirmed positive correlations of PAH content with carbon and nitrogen content.

**Table 1:** The mean value and standard deviation of chemical properties in mull (*n* = 10) and mor (*n* = 10) humus types

Properties	Humus type		*p* value
	Mull	Mor	
pH H_2_O	4.14 ± 0.27	3.81 ± 0.11	n.s.
pH KCl	3.26 ± 0.21	2.90 ± 0.06	*p* < 0.025
C	8.11 ± 4.07	35.98 ± 3.93	*p* < 0.005
N	0.46 ± 0.17	1.47 ± 0.21	*p* < 0.005
C/N	17.1 ± 1.8	24.6 ± 1.70	*p* < 0.005
Y	16.97 ± 5.83	71.13 ± 12.92	*p* < 0.005
Ca	2.04 ± 1.07	4.91 ± 1.53	*p* < 0.020
K	0.38 ± 0.11	0.69 ± 0.19	*p* < 0.020
Mg	0.41 ± 0.16	0.95 ± 0.24	*p* < 0.005
Na	0.03 ± 0.01	0.09 ± 0.03	*p* < 0.005
BC	2.87 ± 1.29	6.65 ± 1.77	*p* < 0.005
Cd	1.34 ± 0.45	3.82 ± 0.58	*p* < 0.005
Cu	20.88 ± 10.23	73.56 ± 11.16	*p* < 0.005
Ni	9.98 ± 2.62	22.42 ± 3.83	*p* < 0.005
Pb	111.21 ± 52.04	389.83 ± 57.76	*p* < 0.005
Zn	68.43 ± 20.16	174.41 ± 26.74	*p* < 0.005
DHA	41.12 ± 26.51	7.99 ± 4.42	*p* < 0.005

Dehydrogenase (DHA) activity is in μmol of triphenyl formazan (TPF) kg^−1^ h^−1^. C and N are shown as %. Y is the hydrolytic acidity in cmol (+) kg^−1^. Ca, Mg, K, and Na content in cmol(+) kg^−1^. Cd, Cu, Ni, Pb and Zn in mg·kg^−1^. *BC* base cations, *n.s.* not significant; *p* values of two-sample *t* tests are presented. Significantly different values *p* < 0.05 are highlighted

**Table 2:** Correlations between PAHs content and humus characteristics

	DHA	pH H_2_O	pH KCl	C	N	C/N	Cd	Cu	Ni	Pb	Zn
PAHs	− 0.56*	− 0.58*	− 0.58*	0.75*	0.73*	0.65*	0.71*	0.70*	0.64*	0.71*	0.69*

*p < 0.05

## Discussion

The study confirmed the strong pollution of the surface soil horizons of the examined area. Regardless of the type of organic matter, high levels of PAHs and heavy metals were noted. According to the scale of pollution proposed by Maliszewska-Kordybach (1996), the mull-type humus samples analyzed in this study are poorly polluted with polycyclic aromatic hydrocarbons. Their content ranges from 200 to 600 μg kg^−1^. In the case of mor-type humus, very strong PAH contamination was noted (sum of PAHs > 1000 μg kg^−1^). High content of PAHs in mor humus, less decomposed, can be explained by the high affinity of hydrophobic organic pollutants to this type of organic matter. Soil organic matter strongly absorbs a significant part of hydrophobic compounds reducing their bioavailability and ensuring their durability in the environment (Maliszewska-Kordybach et al. 2000). The relationship between the amount of PAHs and the amount of organic matter was confirmed by a strong correlation of C content and the sum of PAHs of the examined types of humus. The examined types of humus differ in the content and quality of soil organic matter. The content of PAHs in soils depends on the content and the quality of organic matter to a significant degree, thus showing a high sorption potential (Chen et al. 2007; Pan et al. 2006; Yang et al. 2010a). Different degree of organic matter decomposition in the examined samples was confirmed by the C/N ratio. A more efficient decomposition is related to mull-type humus (lower C/N ratio), while slower decomposition to mor-type humus (higher C/N ratio). Obrist et al. (2015) noted a strong correlation between the OPAH content and the C/N ratio showing strong accumulation of OPAHs in old, decomposed carbon fraction, in support of high persistency of OPAHs in forest soils.

In the study conducted by Maliszewska-Kordybach et al. (2009), the region in which we located the study areas is strongly contaminated by PAHs, especially four to six ring aromatic hydrocarbons compared to other areas of Poland. In our study, we confirmed the very high contents of four, five, and six ring aromatic hydrocarbons, especially in mor-type humus samples. High content of high molecular weight (HMW) of PAHs in mor-type humus compared to mull-type humus indicates low level of their biodegradation. Strongly adsorbed pollutants demonstrate a limited access for soil microorganisms, which slows down degradation processes (Oleszczuk 2007). Our results confirm that mor-type humus strongly adsorb pollutants, especially PAHs. Weaker degradation of hydrocarbons is associated with lower biological activity of the soils. Mull-type humus showed high biochemical activity confirmed by high activity of dehydrogenase, which is often used in ecotoxicological research (Maliszewska-Kordybach et al. 2000; Klimkowicz-Pawlas and Maliszewska-Kordybach 2003; Klamerus-Iwan et al. 2015). Inhibited activity of dehydrogenases detected during the experiment may be the result of direct toxic effects of PAHs on the microorganisms. In addition, the concentration of heavy metals could have a limiting effect on the activity of soil microorganisms in the study conducted. According to Brookes (1995), Pająk et al. (2016), dehydrogenase activity can be used as an indicator of heavy metal contamination of soil. According to Kabata-Pendias (2011), the admissible content of Cd in soil amounts to 1 mg kg^−1^, Cu to 30 mg kg^−1^, Ni to 20 mg kg^−1^, Pb to 50 mg kg^−1^, and Zn to 100 mg kg^−1^. The heavy metals can be toxic for plants above the presented levels. In our study, the permissible content of heavy metals was exceeded in mor humus type. The highest exceedances concern lead and cadmium. Metal pollution has been shown to affect microbial functional diversity (Stefanowicz et al. 2008). Heavy metals are considered as inhibitors of enzymatic and microbial activity in the soil, causing the changes in soil microflora composition and in activity of individual enzymes, which in a consequence leads to a weakening of organic matter decomposition. Variability of soil properties, especially pH, significantly influences the mobility and potential availability of heavy metals. Mor-type humus is characterized by a lower pH, thus creating better conditions for high availability of metals. The mobility of heavy metals in the soil environment is determined by the forms of their occurrence and the mechanisms of their binding to organic and inorganic soil components. In our study, we recorded a relationship between the content of PAHs and heavy metals in the examined types of forest humus. Similar relations in soil samples were recorded by Sun et al. (2014). The correlations between PAHs and heavy metals produced from industrial emissions indicate the source of PAHs and metals in soils (Musa et al. 2010; Fabietti et al. 2010; Sun et al. 2014).

## Conclusion

The forest humus type greatly affects PAH concentrations. The highest content of PAHs and heavy metals was recorded in mor humus type which is less decomposed. Our experiment provided evidence that the quantity and quality of soil organic matter plays an important role in controlling PAH amounts. The mull humus type showed lower content of PAHs and at the same time the highest biological activity confirmed by high dehydrogenase activity. The soil dehydrogenase activity was significantly reduced by PAHs and heavy metal pollution. The investigated humus types are characterized by variability of properties, especially pH which significantly influences the mobility and potential availability of heavy metals.
